# An ApiAP2 Family Transcriptional Factor PfAP2-06B Regulates Erythrocyte Invasion Indirectly in *Plasmodium falciparum*

**DOI:** 10.3390/pathogens14111076

**Published:** 2025-10-22

**Authors:** Qiyang Shi, Kai Wan, Yifei Gong, Jiayao Pang, Yaobao Liu, Jianxia Tang, Qingfeng Zhang, Jun Cao, Li Shen

**Affiliations:** 1 Public Health Research Center, Jiangnan University, Wuxi 214122, China; ryansqy@outlook.com (Q.S.); tangjianxia@jipd.com (J.T.); 2 National Health Commission Key Laboratory of Parasitic Disease Control and Prevention, Jiangsu Provincial Key Laboratory on Parasite and Vector Control Technology, Jiangsu Institute of Parasitic Diseases, Wuxi 214101, China; 3 Key Laboratory of Pathogen-Host Interaction, Ministry of Education, State Key Laboratory of Cardiology and Research Center for Translational Medicine, Shanghai East Hospital, Clinical Center for Brain and Spinal Cord Research, School of Medicine, Tongji University, Shanghai 200120, China

**Keywords:** malaria, *Plasmodium falciparum*, erythrocyte invasion, ApiAP2 transcription factor

## Abstract

Obligate intracellular parasites must efficiently invade host cells to complete their life cycle and facilitate transmission. For the malaria-causing parasite *Plasmodium falciparum*, the invasion of an erythrocyte is a critical process, and thereby a key target for intervention strategies. In this study, we investigate the role of the ApiAP2 family transcription factor PfAP2-06B (PF3D7_0613800) in the intraerythrocytic developmental cycle of *P. falciparum* and focus on its regulation of genes involved in erythrocyte invasion. Conditional knockdown of PfAP2-06B resulted in a defect in asexual growth and impaired erythrocyte invasion. Bulk RNA sequencing (RNA-seq) analysis revealed that PfAP2-06B modulates the expression of invasion-related genes during the schizont stage. Single-cell RNA sequencing indicated that PfAP2-06B influences invasion gene expression and contributes to stochastic variations in expression of cell-to-cell genes. These results underscore the critical function of PfAP2-06B in the process of erythrocyte invasion and suggest its potential as a target for novel malaria control strategies. Importance: Understanding gene regulation in *Plasmodium falciparum* is essential for uncovering mechanisms of parasite development and pathogenicity. The research underscores the pivotal role of PfAP2-06B in regulating critical aspects of Plasmodium intraerythrocytic development and host cell invasion, demonstrating that PfAP2-06B plays a key role in orchestrating stage-specific gene expression. These findings provide new insights into the transcriptional networks of P. falciparum and highlight PfAP2-06B as a potential target for therapeutic intervention. This work advances our understanding of malaria pathogenesis and developing effective interventions.

## 1. Introduction

The protozoan phylum Apicomplexa encompasses a diverse group of obligate intracellular parasites, many of which are of medical and veterinary importance [1]. Among these, Plasmodium falciparum is a prominent causative agent of human malaria, a disease that remains a major global health challenge, with 249 million malaria infections cases and 608,000 deaths in 2022 [2,3]. The life cycle of *P. falciparum* is complex, and involves both vertebrate and insect hosts; the intraerythrocytic developmental cycle (IDC) in the human host is central to disease pathogenesis [4].

In the human host, *P. falciparum* begins its asexual replication cycle with the invasion of erythrocytes by merozoites. This marks the start of the 48 h IDC, during which the parasite undergoes significant morphological and metabolic changes [5,6]. After erythrocyte invasion, the merozoite develops into a trophozoite, which metabolizes hemoglobin and acquires nutrients from serum. The intraerythrocytic parasite progresses through a series of developmental stages and, ultimately, to the schizont stage, during which the parasite replicates asexually via schizogony.

The division of the parasite nucleus and cytoplasm during schizogony results in 16 to 32 daughter merozoites, which are packaged within the schizont and are then released into the bloodstream following erythrocyte rupture [7]. Merozoites are equipped with a specialized set of proteins that are essential for a new round of erythrocyte invasion, including surface proteins such as merozoite surface proteins (MSPs), and micronemal and rhoptry organellar secreted proteins, which facilitate the recognition and binding of new erythrocytes [8,9,10]. The process of egress and invasion repeats cyclically, thus increasing the parasite burden in the bloodstream and resulting in the characteristic clinical symptoms of malaria, including fever, chills, and anemia.

Understanding the molecular and cellular mechanisms underlying the egress and invasion processes is crucial for developing new therapeutic strategies against P. falciparum and malaria. Despite significant advances in malaria treatment and prevention, the parasite’s complex life cycle and ability to rapidly evolve resistance to drugs pose ongoing challenges to controlling the disease [11]. Moreover, critical proteins that are exposed to the host immune system are typically expanded into antigenically variant gene families [12]. Therefore, elucidating the factors that regulate merozoite egress, invasion, and the subsequent immune responses is critical for identifying novel drug targets and vaccine candidates that may help in the fight against malaria [13,14].

In *P. falciparum*, epigenetic regulation through histone modifications and histone variants plays a central role in the control of gene expression [15]. The regulation of transcription in eukaryotes is influenced by specific transcription factors (TFs), which orchestrate developmental transcriptional programs [16,17]. Twenty-seven AP2 (ApiAP2) DNA-binding proteins unique to Apicomplexa have been characterized as central regulators of transcription, playing essential roles in developmental transitions, cellular differentiation, and environmental response mechanisms [15,18,19]. Among the ApiAP2 family proteins, the transcription factor AP2-I has been shown to be critical for erythrocyte invasion, suggesting its involvement in the regulation of genes associated with the invasion process [20]. The binding of AP2-I occurs prior to the recruitment of the bromodomain protein PfBDP1, which plays a critical role in the regulation of invasion-related genes. Another ApiAP2 TF, PF3D7_0613800, has been implicated in invasion, with its expression peaking during the late trophozoite to schizont stage. PF3D7_0613800 was identified as an interaction partner of PfBDP1 through mass spectrometry [21], and pull-down assays using a novel DNA motif identified by ATAC-seq [22] further support the interaction between PF3D7_0613800, AP2-I, and PfBDP1.

In this study, we employed CRISPR/Cas9 genomic editing technologies to investigate the function of PF3D7_0613800 throughout the *Plasmodium* IDC [23]. Using inducible gene knockdown, we confirm that PF3D7_0613800 encodes a *Plasmodium*-specific ApiAP2 transcription factor [24], which we designate as PfAP2-06B. Our results show that PfAP2-06B is essential for parasite growth, development, and invasion. Through growth curve assays, bulk RNA sequencing (RNA-seq), and single-cell RNA sequencing (scRNA-seq) analysis [25], we establish that PfAP2-06B regulates the expression of genes linked to invasion, with a specific expression pattern during the schizont phase of the intraerythrocytic life cycle.

## 2. Methods

### 2.1. Parasite Cultures

The *Plasmodium falciparum* 3D7 strain was grown in type O fresh human erythrocytes, following the described protocol, using RPMI 1640 medium (Gibco, Waltham, MA, USA) containing 0.5% Albumax I (Invitrogen, Waltham, MA, USA). Cultures were incubated at 37 °C under a gas mixture of 5% CO_2_, 5% O_2_, and 90% N_2_ at 37 °C [26]. Parasites were routinely synchronized at the ring stage using repeated 5% sorbitol treatments. For growth curve assays, a 5 h synchronization window was achieved by purifying schizonts with Percoll–sorbitol gradients (70% and 40% Percoll), followed by a 5% sorbitol treatment 5 h afterward [27].

### 2.2. Plasmid Construction

The pL6cs-AP2-06B-2xfkbp-gfp-2xfkb plasmid was constructed according to the previously described method [23,24,27]. The guide RNA was annealed using complementary oligonucleotides and ligated into the pL6cs plasmid between Xho I and Avr II sites. The C-terminal fragment of pfAP2-06B with 2×fkbp-gfp-2×fkbp was then inserted into Afl II and Asc I sites. The plasmid was verified by sequencing, transformed into *Escherichia coli* XL10 for amplification, and purified. Primers used for construction are listed in Supplementary Table S1.

### 2.3. Generation of Transgenic Parasite Lines

Transfections were conducted in uninfected erythrocytes using 100,000 ng of purified pL6cs-pfap2-06b-2xfkbp-gfp-2xfkbp plasmid, along with 100,000 ng of pUF1-Cas9-DSM1, followed by the addition of purified schizont stage 3D7 parasites. Parasites were cultured in the presence of 2.5 nM WR99210 and 1.5 μM DSM1 (Invitrogen), until live parasites were observed 3 weeks later in thin blood smears stained with Giemsa solution. The sequences at the designed integration sites were verified by PCR amplification of genomic DNA, followed by sequencing to confirm genetic editing. Transfections were then performed in uninfected erythrocytes using 100,000 ng of purified pLyn-FRB-mCherry plasmid and 2 μg/mL BSD, with the addition of schizont-stage PfAP2-06B-2xFKBP-GFP-2xFKBP parasites. Clonal lines of transgenic PfAP2-06B-2xFKBP-GFP-2xFKBP::pLyn-FRB-mCherry (PfAP2-06B-KS) parasites were isolated by limiting dilution cloning. Primers used for verification are listed in Supplementary Table S2.

### 2.4. Growth Curve Assays

Parasites were tightly synchronized to a 5 h window and plated at 0.1% parasitemia with 2% hematocrit in a 6-well plate. PfAP2-06B-KS parasites were divided into two 2 mL dishes, one with 250 nM Rapa and the other as a control (EtOH of the same volume as Rapa). Parasitemia levels were monitored over the next three cycles by counting trophozoites in Giemsa-stained smears.

### 2.5. Immunofluorescence Assays

Immunofluorescence assays were performed to localize PfAP2-06B as described [28]. Schizont-stage parasites were fixed with 4% paraformaldehyde for 10 min, washed with PBS, and incubated with primary antibodies against GFP (1:500–1:1000) and mCherry (1:300). Secondary antibodies, AlexaFluor 488 goat anti-mouse IgG (1:500) and AlexaFluor 568 goat anti-rabbit IgG (1:500), were applied. For nuclear localization, samples were incubated with DAPI for 30 min. Preparations were visualized using a Nikon A1R microscope (Nikon, Tokyo, Japan) at 60× to 100× magnification, and images were acquired with NIS Elements software 4.2.0 and processed with Adobe Photoshop CS6 Extended v13.0.0.0.

### 2.6. Merozoite Counting Assay

To quantify the number of nuclei per schizont, highly synchronized parasites were cultured with or without knockdown (KD) drugs throughout their respective life cycles. The number of merozoites produced per schizont was assessed in mature segmenters. Schizont nuclei were Giemsa-stained, and the number of nuclei was counted in 26 mature schizonts.

### 2.7. Merozoite Release Assay

PfAP2-06B-KS parasites were sorbitol-synchronized as 0–3 h rings with 1% parasitemia, plated in replicate (three replicates for [+]Rapa and [−]Rapa controls; three replicates for [+]Rapa and [−]Rapa controls each with the addition of 200 nM ML-10 [29] at the trophozoite stage). Data are presented as mean +/− S.D. (or data range for N = 3 samples). At the 46, 48, and 50 h stages, calculate the schizont parasitemia using microscopy with Giemsa-stained blood smears (FANYIGX, Beijing, China).

### 2.8. Invasion Assays

100 μL erythrocytes were pretreated with 1 mg/mL trypsin at 37 °C for 1 h under gentle shaking, then washed twice with incomplete medium. Soybean trypsin inhibitor was subsequently added to the packed erythrocytes, which were incubated at room temperature for 10 min. Following three washes with medium, the erythrocytes were resuspended in an equal volume of medium and stored at 4 °C until use.

Invasion assay was performed to determine the invasion efficiency for proposed transgenic parasite lines. Briefly, mature schizonts were purified by centrifugation on 40%/70% Percoll gradient. Enriched schizont-infected erythrocytes were counted, and 8 × 10^5^ schizonts were mixed with trypsin-treated erythrocytes at a 1:50 ratio in 200 μL complete medium, pH 7.2 (RPMI 1640, 24 mM HEPES, 360 μM hypoxanthine, 24 mM NaHCO_3_, 10 μg of gentamicin per mL, and 5% Albumax [GIBCO]), gassed with a 5% CO_2_, 5% O_2_, and 90% N_2_ mixture. Mixtures were added into 96-well plates with duplicate wells for each treatment. After incubation under standard conditions for 20 h, thin blood smears were prepared. Invasion efficiency was determined by microscopically counting ring-stage parasites, with at least 50 fields of the smear examined. Invasion efficiency was calculated by dividing the parasitemia after invasion by the pre-invasion baseline parasitemia.

### 2.9. Bulk RNA-Seq

PfAP2-06B-2xFKBP-GFP-2xFKBP::pLyn-FRB-mCherry parasites were synchronized to a 5 h window with or without 250 nM Rapa treatment. At 38 to 42 h post-invasion (HPI), samples were collected during the schizont stage in TRIzol (two biological replicates). Total RNA was extracted following the manufacturer’s protocol (Zymo Research, Irvine, CA, USA). For strand-specific RNA-seq, poly(A) selection was performed using KAPA mRNA Capture Beads, followed by fragmentation to approximately 300–400 nucleotides. Library preparation was completed using the KAPA Stranded mRNA-Seq Kit (KK8421) (KAPA Biosystems, Wilmington, MA, USA), and sequencing was conducted on an Illumina HiSeq XTen system to generate 150 bp paired-end reads.

### 2.10. Bulk RNA-Seq Analysis

Low-quality and adaptor sequences were removed from the reads using Cutadapt (v1.16) with appropriate parameters. RNA sequencing reads were then aligned to the *Plasmodium falciparum* 3D7 genome (Pf 3D7 v45, obtained from PlasmoDB) using HISAT2 (v2.1.0) [30] in strand-specific mode (--rna-strandness RF). Mapped reads were assembled into transcripts based on the PlasmoDB GFF annotation files (Pf 3D7 v45) using featureCounts (v1.6.4) [31] with the following parameters: -M -p -B -C -s 2. Gene expression was quantified as fragments per kilobase of transcript sequence per million read pairs mapped (FPKM), using featureCounts output in R version 4.1.3. Differential gene expression was determined with a fold change threshold of >1.5 and a *p*-value < 0.05. Gene Ontology (GO) analysis of differentially expressed genes was performed using clusterProfiler, with a BH-adjusted *p*-value threshold of <0.01.

### 2.11. Single-Cell Suspension Preparation

Schizonts, with or without Rapa treatment, were collected by 40%/70% Percoll from culture and resuspended in 2–3 mL of 1x red blood cell lysis buffer (Invitrogen, 00-4333), then incubated at room temperature for 2–4 min. Cell viability was assessed using trypan blue exclusion, with a required viability of >85%. Single-cell suspensions were counted using a hemocytometer or Countess II Automated Cell Counter, and the concentration was adjusted to 400–1200 cells/μL.

### 2.12. GEM Generation and 3′Gene Expression Library Construction

Single-cell suspensions were prepared and loaded onto the 10× Chromium platform following the manufacturer’s instructions for the 10× Genomics Chromium Single-Cell 3′ Kit (V3) (10× Genomics, Pleasanton, CA, USA). cDNA amplification and library construction were carried out using the standard protocol. Sequencing of the libraries was performed on an Illumina NovaSeq 6000 system (paired-end, 150 bp multiplexed run) by Shanghai Personal Biotechnology (Shanghai, China).

### 2.13. Single-Cell RNA-Seq Data Processing

Our study analyzed two *Plasmodium falciparum* single-cell RNA-seq datasets comprising 23,683 cells from the Rapa treatment group (mean 12,747 reads/cell) and 25,505 cells from the EtOH control group (mean 14,344 reads/cell), with 5536–5538 genes detected per sample. Initial processing using Cell Ranger (v8.0.0) generated raw expression matrices that were subsequently integrated through Seurat’s merge function (v4.1.0) with cell barcode prefixes distinguishing treatment groups. Doublet removal was performed using DoubletFinder prior to quality control filtering that excluded cells with <500 UMIs, <100 detected genes, >0.25% apicoplast-derived transcripts (PF3D7-API), or >2% mitochondrial gene expression (PF3D7-MIT). SCTransform normalization addressed sequencing depth variation while preserving biological heterogeneity, followed by selection of 1500 highly variable genes for dimensionality reduction. Principal component analysis with 12 PCs informed the construction of a shared nearest neighbor graph for Louvain clustering (resolution = 0.5). Uniform Manifold Approximation and Projection (UMAP) visualization in 2D space revealed distinct transcriptional states that were annotated into six developmental stages (early/late rings, trophozoites, and schizonts) based on established Plasmodium stage-specific markers through manual cluster merging. The final dataset retained high-quality single cells with clear cell cycle progression characteristics for downstream comparative analysis [32].

For temporal mapping of Plasmodium developmental stages, we integrated bulk RNA-seq time course data (24 timepoints spanning 4–52 h post-infection) with single-cell transcriptomes. To enhance cluster biological coherence, we performed harmonic integration using Harmony (30 harmony dimensions) with Leiden clustering at resolution = 2 in the corrected space. UMAP visualization utilized the first 30 harmony dimensions to preserve developmental continuum topology.

Temporal alignment employed dynamic time warping with extended Jaccard distance metric, comparing each cluster’s expression vector against standardized bulk RNA-seq profiles from synchronized parasites. Developmental timing assignments were validated through concordance checks between computationally predicted timepoints and manually annotated cell cycle stages.

## 3. Results

### 3.1. PfAP2-06B Is Essential for Plasmodium Falciparum Parasite Growth and Development

**Figure 1:**
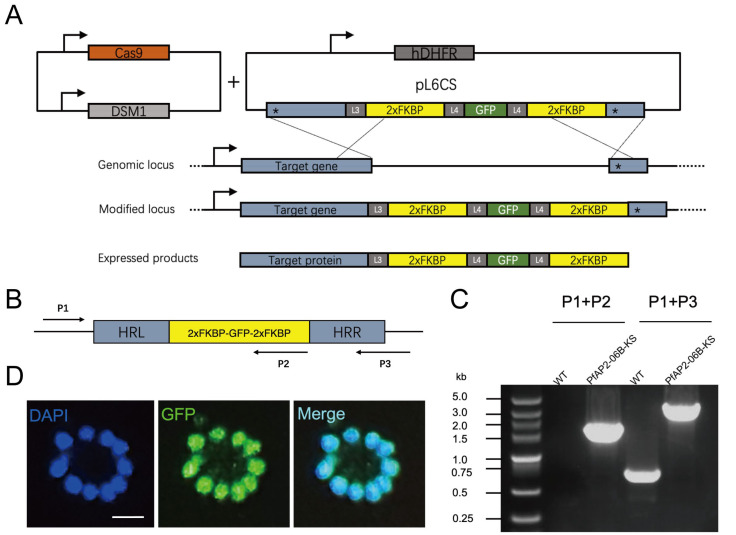
Schematic of the plasmid and verification of PfAP2-06B-2xFKBP-GFP-2xFKBP transgenic parasite lines. (**A**) Generation of a PfAP2-06B transgenic parasite line tagged with 2xFKBP-GFP-2xFKBP at the C-terminus using the CRISPR/Cas9 gene editing system. ‘*’ denotes homologous sequences, arrow indicates the transcription direction. (**B**) Schematic diagrams for verification primers. Arrows indicate the direction of the primers. (**C**) The 2xFKBP-GFP-2xFKBP tagged PfAP2-06B transgenic line was verified by diagnostic PCR using P1, P2, and P3 primers. (**D**) Representative fluorescence microscopy images of PfAP2-06B-2xFKBP-GFP-2xFKBP late stage schizonts. Immunofluorescence assays using anti-GFP revealed perinuclear distribution of PfAP2-06B in the 3D7/PfAP2-06B-2xFKBP-GFP-2xFKBP line.

PfAP2-06B (PF3D7_0613800) is the largest transcription factor in the *P. falciparum* ApiAP2 family and contains three predicted DNA-binding domains [33]. The orthologue of PF3D7_0613800 in the rodent malaria parasite, *Plasmodium berghei* (PBANKA_0112100), was shown to be refractory to domain deletion, indicating that this predicted transcription factor might be crucial for parasite growth and development during the IDC [34]. To investigate the function of PF3D7_0613800, we endogenously tagged the C-terminal of PfAP2-06B with 2xFKBP-GFP-2xFKBP. A GFP tag between the two FKBPs allowed localization of the native target (Figure 1A). Following 20 days of culture and drug selection, we successfully obtained a 3D7/PfAP2-06B-2xFKBP-GFP-2xFKBP transgenic parasite line. Genomic DNA was extracted from the transgenic parasites, and two sets of PCR primers were utilized to confirm the accurate editing of the *pfap2-06b* locus (Figure 1B,C). Fluorescence microscopy was employed to investigate the expression levels and subcellular localization of the PfAP2-06B-2xFKBP-GFP-2xFKBP fusion protein. Similarly to other ApiAP2 transcription factors, PfAP2-06B localizes to the nucleus (Figure 1D).

**Figure 2:**
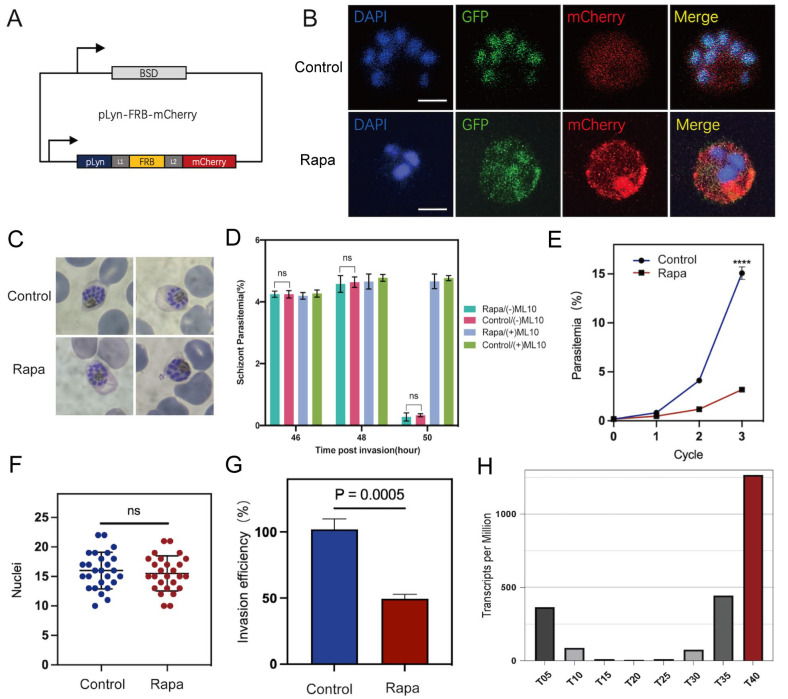
PfAP2-06B is essential for parasite growth and invasion. (**A**) Schematic of the pLyn-FRB-mCherry plasmid. (**B**) Representative fluorescence microscopy images of PfAP2-06B-2xFKBP-GFP-2xFKBP::pLyn-FRB-mCherry late stage schizonts. Immunofluorescence assays (IFA) using anti-GFP revealed perinuclear distribution of PfAP2-06B in the 3D7/PfAP2-06B-2xFKBP-GFP-2xFKBP line using anti-mCherry revealed pLyn-FRB-mCherry. The first panel was treated as the Control (RAPA−), and the second panel was treated with RAPA (RAPA+). (**C**) Giemsa-stained blood smears after three life cycles of synchronized parasites treated with Rapa and ethanol as control. (**D**) Quantification of schizont at the 46, 48, and 50 h stages using microscopy with Giemsa-stained blood smears, to assess the schizonts rupture normally. No difference in Schizont Parasitemia (%) was observed between control (RAPA−) and treated (RAPA+). (**E**) Growth curves of PfAP2-06B-2xFKBP-GFP-2xFKBP::pLyn-FRB-mCherry in the presence or absence of Rapa over three growth cycles (three biological replicates, average parasitemia ± s.d.). Comparisons between control (RAPA−) and treated (RAPA+) schizonts were performed with Student’s *t*-test. **** indicates *p*-value < 0.0001. (**F**) Nuclei in control (RAPA−) and treated (RAPA+) schizonts. *p* value of 0.556, unpaired *t* test; error bar is mean with ± standard deviation (s.d.). n.s., not significant. (**G**) Invasion efficiency of one life cycle’s control (RAPA−) and treated (RAPA+) 2xFKBP-GFP-2xFKBP::pLyn-FRB-mCherry parasites (three biological replicates, average parasitaemia ± s.d.). (**H**) Expression profiles of PfAP2-06B in the *P. falciparum* 3D7 strain throughout the intraerythrocytic developmental cycle according to RNA-seq transcriptomic analysis [22].

To create a knockdown parasite line, we introduced a plasma membrane mislocalizer, pLyn-FRB-mCherry, into the transgenic parasite line PfAP2-06B-2xFKBP-GFP-2xFKBP to create a line hereafter referred to as PfAP2-06B-KS (Figure 2A). In the absence of Rapa, the FRB and FKBP domains do not interact. However, upon the addition of Rapa, which binds to both FRB and FKBP, dimerization of these domains is induced. This interaction promotes the translocation of PfAP2-06B from the nucleus to the plasma membrane, ultimately leading to its degradation or reduction. As demonstrated by fluorescence microscopy, the introduction of Rapa to cultures of PfAP2-06B-KS parasites results in the translocation of PfAP2-06B from the nucleus to the plasma membrane. In contrast, in the absence of Rapa, PfAP2-06B remains predominantly localized in the nucleus (Figure 2B).

To evaluate the role of PfAP2-06B in parasite growth and development, the inducible knockdown line was tightly synchronized within a 5 h window and cultured in the presence or absence of Rapa [35]. Parasite progression was monitored across three consecutive blood-stage cycles using Giemsa-stained thin blood smears: at which time in the presence of Rapa the PfAP2-06B-2xFKBP-GFP-2xFKBP::pLyn-FRB-mCherry parasites still maintained normal morphology as assessed by microscopy (Figure 2C). The quantification of schizonts at the 46, 48, and 50 h stages was performed using microscopy with Giemsa-stained blood smears to assess the normal rupture of schizonts (Figure 2D). To serve as a control, 200 nM of ML-10 was added to the trophozoites immediately prior to merozoite egress, effectively arresting P. falciparum growth [29]. The Rapa pool demonstrated normal schizont rupture, similar to the control, indicating no significant difference in rupture behavior between the two groups. However, compared to the control group, the knockdown of PfAP2-06B resulted in a greater than 70% reduction in parasite-infected erythrocytes (unpaired *t*-test, *p*-value < 0.0001; Figure 2E), indicating that PfAP2-06B is essential for the blood-stage growth and development of *P. falciparum*, but does not affect parasite morphology.

### 3.2. PfAP2-06B Modulates Parasite Invasion of Erythrocytes

To determine the underlying cause of the decreased number of infected erythrocytes following PfAP2-06B knockdown, we performed a merozoite counting assay after treatment with Rapa. Similarly to the controls, the experimental parasites developed into morphologically normal segmented schizonts, and there was no statistically significant difference in the number of nuclei in late schizonts of PfAP2-06B-2xFKBP-GFP-2xFKBP::pLyn-FRB-mCherry parasites treated by Rapa (Figure 2F).

To evaluate the efficiency of erythrocyte invasion, we collected strictly synchronized late-stage schizonts, and introduced them into an equal volume of erythrocytes [36], both with and without Rapa treatment for one life cycle. The invasion efficiency was observed to decrease by approximately 40% following PfAP2-06B knockdown, which suggests that PfAP2-06B plays a role in parasite invasion of erythrocytes (Unpaired *t*-test, *p*-value = 0.0028, Figure 2G).

### 3.3. PfAP2-06B Impacts Egress and Invasion Gene Transcription at the Schizont Stage

**Figure 3:**
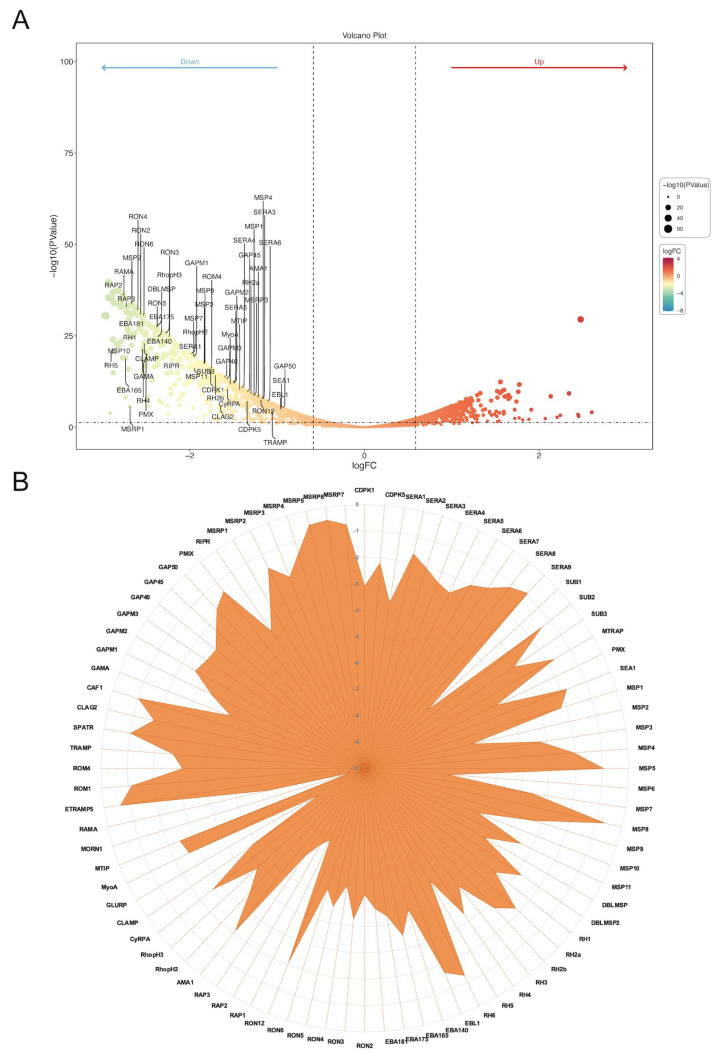
PfAP2-06B impacts egress and invasion-related genes transcription. (**A**) Transcriptome changes in the PfAP2-06B knockdown schizonts (fold change of >2 and false discovery rate of <0.05). (**B**) Expression of 84 invasion-related gene transcripts upon AP2-06B knockdown, the vertical axis indicates the degree of decrease.

**Figure 4:**
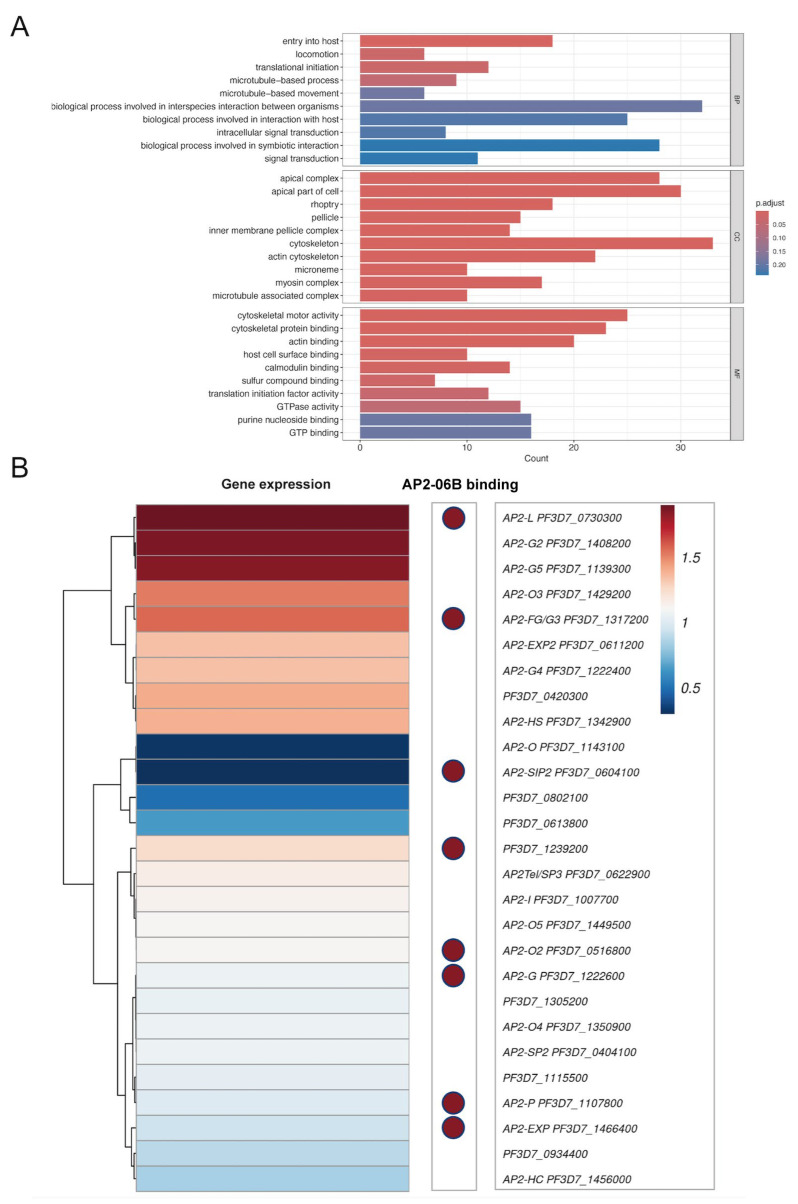
PfAP2-06B knockdown transcriptome analysis. (**A**) Gene ontology (GO) analysis pathways and functional families enriched in PfAP2-06B-2xFKBP-GFP-2xFKBP::pLyn-FRB-mCherry heterochromatic target genes at the schizont stage (BH adjusted enrichment *p*-values of <0.01), BP (Biological Process), CC (Cellular Component) and MF (Molecular Function). (**B**) PfAP2 gene expression following PfAP2-06B knockdown and PfAP2-06B binding distribution at AP2 gene locus.

Time-series expression profiling of the IDC in the *P. falciparum* 3D7 strain revealed that the PfAP2-06B mRNA transcript level reached its peak during the schizont stages (Figure 2H) [22]. To investigate the role of PfAP2-06B during this stage, transcriptome analyses were performed on the PfAP2-06B-2xFKBP-GFP-2xFKBP::pLyn-FRB-mCherry parasites. Parasites were tightly synchronized within a 5 h window and cultured with or without Rapa. After reinvasion, parasites were harvested for bulk RNA-seq at 40 to 45 HPI. Analyses revealed significant effects of PfAP2-06B on gene transcription at this timepoint, with 404 genes downregulated by more than twofold. Given the pronounced decrease in erythrocyte invasion efficiency, we focused on 84 genes which are associated with egress and invasion (Figure 3A) [8], among which 60 genes were downregulated by more than two-fold (Figure 3B). The knockdown of AP2-06B disrupted the broader transcriptional network governing invasion and pathogenesis. Gene ontology (GO) analysis revealed that AP2-06B regulates a set of genes involved in invasion (Figure 4A), which is consistent with the results of the invasion efficiency assay, as well as genes implicated in cell adhesion, motility, and membrane trafficking. The disruption of these interconnected pathways likely contributes to the observed invasion defect and the altered developmental progression of the parasite.

ChIP-seq analysis of PfAP2-06B indicated that the protein bound to the promoters of several egress and invasion genes that were downregulated in the PfAP2-06B knockdown strain (Supplementary Figure S1) [27], suggesting that PfAP2-06B directly regulates these genes. However, it did not bind to the promoters of most of the identified genes, indicating that the effects of PfAP2-06B knockdown on transcription are likely both direct and indirect. Altogether, the data suggest that PfAP2-06B is crucial for the expression of genes associated with egress and invasion.

### 3.4. PfAP2-06B Impacts the Transcription of Apiap2s and Binds to the Promoters of Eight Other Apiap2 Genes

The AP2 family of *Plasmodium* transcription factors are an important system for regulating stage-specific gene expression. The cooperation of AP2 transcription factors is a critical aspect of gene regulation in *P. falciparum* and drives the coordination of developmental stages and ensures the proper expression of genes required for parasite survival and transmission. The *Plasmodium* genome encodes a diverse family of AP2-like transcription factors that act in a network, with different members playing specific roles at different stages of the parasite life cycle. These factors often cooperate in a highly orchestrated manner to ensure timely gene expression during asexual replication, sexual differentiation, and host cell invasion.

To investigate the relationship between AP2-06B and other AP2 transcription factors, we analyze the expression of various AP2 factors in the AP2-06B knockdown strain, as well as the binding of AP2-06B through ChIP-seq analysis. In the PfAP2-06B knockdown strain, the transcription of Pfapiap2 genes exhibited notable changes; specifically, *ap2-l, ap2-g2,* and *ap2-g5* were significantly upregulated, while *ap2-o* and *ap2-sip2* were markedly downregulated (Figure 4B). PfAP2-06B was found to bind to the promoters of eight PfAP2 genes during the schizont stage (Supplementary Figure S1) [27], indicating that these genes are directly regulated by PfAP2-06B, including *ap2-g*, which plays a key role in gametocytogenesis [37]. Overall, these findings suggest that PfAP2-06B is involved in the regulation of different AP2 factors cooperation.

### 3.5. Differential Expression of Invasion Genes in Schizonts Reveals a Regulatory Role of PfAP2-06B in Modulating Developmental Stage-Specific Variability

Genetically identical cells are known to exhibit phenotypic variability even under the same environmental conditions. Single-cell transcriptomics is a rapidly advancing research tool, and provides a comprehensive and in-depth understanding of biological systems at the single-cell level. Recent studies have identified significant transcriptional variation during the schizont stage, characterized by a distinct set of highly variable invasion-related gene transcripts [38], which contrasts with data obtained from bulk RNA sequencing.

**Figure 5:**
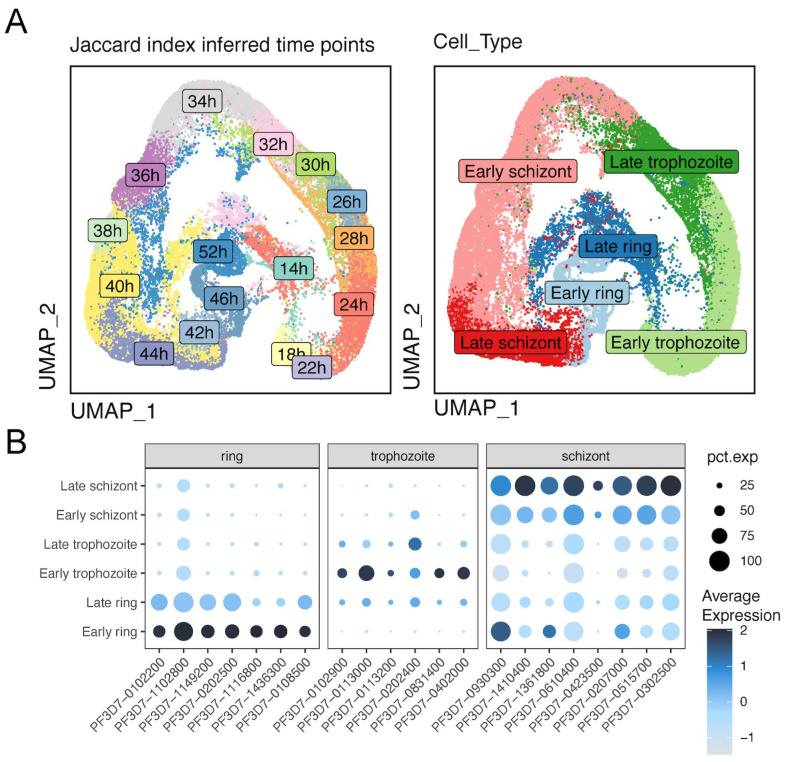
Patterns of gene expression across different life cycle stages. (**A**) Left UMAP plot showing the distribution of subpopulations at different timepoints post-invasion (hpi) with an interval of 2 hr. Right UMAP plot showing the *P. falciparum* asexual development stage distribution. (**B**) Marker genes of ring, trophozoite, and schizont stages. pct.exp represents the percentage of expression.

**Figure 6:**
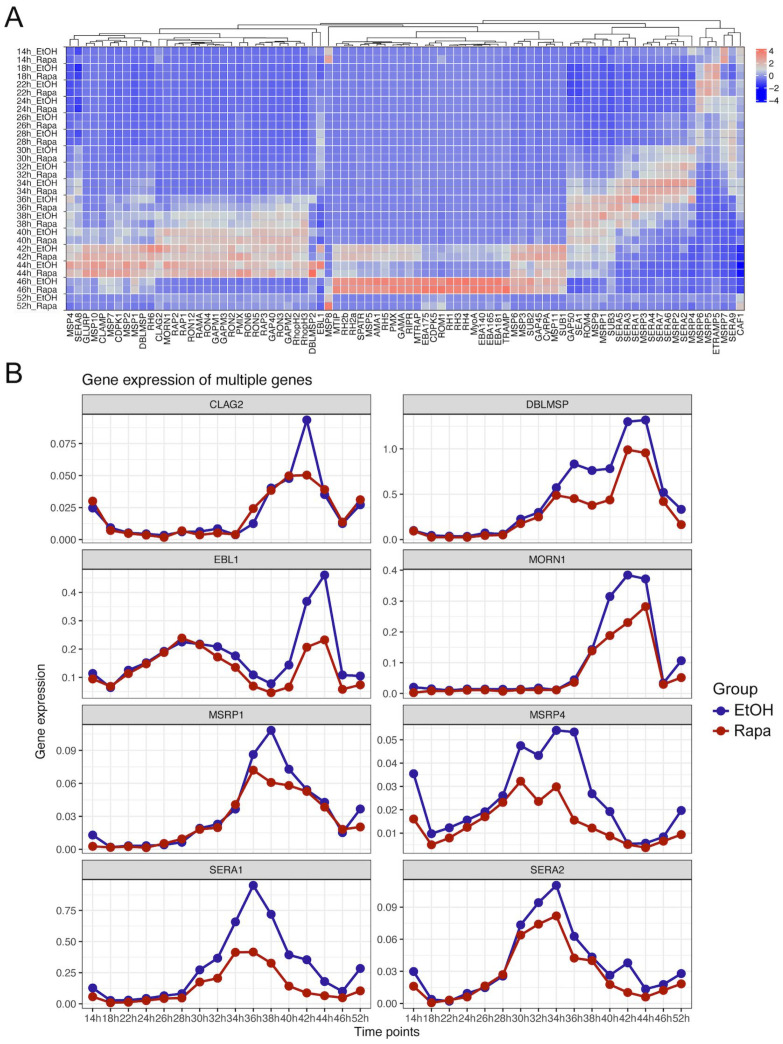
Transcriptional expression of invasion-related genes at the single-cell level following PfAP2-06B Knockdown. (**A**) Expression of 84 invasion-related genes at various timepoints with and without Rapa treatment (**B**) Time-dependent significant downregulation of invasion-related genes expression following PfAP2-06B knockdown.

Single cell methodologies, such as 10x Genomics single-cell RNA sequencing (scRNA-seq), are well suited to compare developmental stages and, thereby, to refine differential transcriptional patterns [39], previously analyzed by “bulk” approaches. We employed 10x Genomics scRNA-seq to investigate the differential expression of invasion genes and to explore the impact of PfAP2-06B knockdown on these processes [40,41]. We focused on late-stage schizonts, to examine the transcriptional variability present in invasion genes arising from developmental differences between individual cells. Single parasites were segregated into 14 to 52 HPI groups (Figure 5A) based on pseudotemporal ordering [32], both for the PfAP2-06B knockdown strain and the control, using specific stage marker genes (Figure 5B). The expression of various invasion gene families exhibited distinct temporal patterns across different HPI groups, with peak expressions of the *msp* and *ron* gene families were observed between 40 and 44 HPI (Figure 6A) [42], while the *eba* and *rh* gene families showed peak expressions primarily at 46 HPI. In contrast, the sera and *msrp* gene families peaked between 34 and 36 HPI. These observations suggest variable expression levels of critical invasion-related gene transcripts in mature schizonts at different developmental stages.

Further comparison of invasion gene expression between the control and PfAP2-06B knockdown strains revealed that several genes, including *ebl1*, *sera1*, and *msrp4*, exhibited a marked decline in expression (Figure 6B) [43]. Additionally, genes such as *clag2*, *dblmsp*, *morn1*, *msrp1*, and *sera2* showed a downward trend in expression. These findings underscore the regulatory role of PfAP2-06B in modulating invasion gene expression and highlight its influence on the stochastic variations observed in cell-to-cell gene expression.

## 4. Discussion

Our study reveals that PfAP2-06B, a key member of the largest transcription factor family in *Plasmodium falciparum*, plays a critical but uncharacterized role in the IDC of the malaria parasite [18]. Our data indicate that PfAP2-06B is crucial for parasite survival during the asexual blood stages, particularly at the schizont stage, and is essential for successful invasion of infected erythrocytes [44]. The inability to generate a PfAP2-06B knockdown strain, combined with the results from large-scale mutagenesis screens, strongly suggests that this transcription factor is crucial for maintaining parasite development and viability during these stages [45].

To investigate the function of PF3D7_0613800, we attempted to generate a ribozyme knockdown system for the gene [46], but it proved inefficient. While ribozymes are an emerging tool for manipulating gene expression in *P. falciparum*, their effectiveness is limited in knocking down larger proteins. Large proteins often play critical roles in complex biological processes, such as host cell invasion, immune evasion, and metabolism [47]. Knocking down such proteins requires highly efficient and specific gene-silencing techniques. Although ribozymes offer some advantages, they may not provide sufficient silencing to induce a phenotypic effect, particularly if the protein is part of a redundant or compensatory pathway. Given that PF3D7_0613800 is a 485 kDa protein [33], we subsequently selected a ‘knock sideways’ system to translocate the protein from the nucleus to the membrane.

At the molecular level, PfAP2-06B functions primarily as a stage-specific transcriptional regulator, with its peak expression occurring during the late trophozoite to schizont transition [22]. This stage-specific expression is consistent with the observed regulatory effects of PfAP2-06B on key genes involved in parasite egress and erythrocyte invasion. Using bulk RNA-seq analysis, we identified a substantial downregulation of approximately 60 genes related to invasion and egress in PfAP2-06B-KS parasites. The functional enrichment of these genes underscores the importance of maintaining the stochastic expression of invasion-related genes, which is likely crucial for successful parasite invasion across variable host cell surface receptors [20]. This is particularly significant given the dynamic nature of host–pathogen interactions during the blood stages of *Plasmodium*. The interaction of PfAP2-06B with other well-characterized transcriptional regulators, such as PfBDP1 and PfAP2-I, as revealed by mass spectrometry, suggests that PfAP2-06B likely operates within a regulatory network that governs invasion and egress events [21]. These interactions further support the hypothesis that the role of PfAP2-06B during erythrocyte invasion is tightly coordinated with other transcriptional factors involved in the parasite life cycle.

The association of PfAP2-06B with drug resistance mechanisms adds another layer of complexity to its role. Notably, mutations in PF3D7_0613800, the gene encoding PfAP2-06B, have been implicated in resistance to quinine and other antimalarial compounds, highlighting its potential involvement in the regulation of drug resistance [48]. This observation aligns with findings from chemo genetic screens, which identified mutations in PF3D7_0613800 as contributing to resistance against several Medicines for Malaria Venture (MMV) compounds [49,50,51]. In our previous work, we investigated the effects of the AP2-06B mutation and its knockdown on resistance to artemisinin and quinoline-based drugs [52]. Additionally, the regulation of PfAP2-06B expression by lysophosphatidylcholine (lysoPC) and PfAP2-G further complicates its role [53], suggesting that PfAP2-06B is responsive to lipid- and protein-mediated signaling pathways that could influence both parasite development and drug resistance. The essential function of PfAP2-06B during the schizont stage and its involvement in key processes such as parasite invasion, combined with its potential role in antimalarial drug resistance, positions it as an attractive target for therapeutic intervention.

In conclusion, this study highlights the crucial role of PfAP2-06B in regulating key aspects of Plasmodium development and host cell invasion. It also suggests that targeting this transcription factor could offer new insights into both therapeutic and prophylactic strategies against malaria. Further research into the detailed mechanisms by which PfAP2-06B regulates gene expression and interacts with other key proteins will be essential for advancing our understanding of malaria pathogenesis and developing effective interventions.

## Data Availability

The Bulk RNA-seq data presented in the study are deposited in the SRA repository, accession number PRJNA1202365. The single-cell RNA-seq data generated in this study have been deposited in the SRA repository under accession number PRJNA1203001. The original contributions are included in the article and Supplementary Material. For further inquiries, please contact the corresponding authors.

## Supplementary Materials

The following supporting information can be downloaded at: https://www.mdpi.com/article/10.3390/pathogens14111076/s1, Figure S1: PfAP2-06B occupancy at representative apiap2 genes and invasion genes; Table S1: Primers used for construction and coding sequence; Table S2: Primers used for verification.
