# Supplementary material for: An ApiAP2 Family Transcriptional Factor PfAP2-06B Regulates Erythrocyte Invasion Indirectly in Plasmodium falciparum
Source: Pathogens. 2025 Oct 22;14(11):1076. doi: 10.3390/pathogens14111076 (PMC12655549; doi:10.3390/pathogens14111076)
Supplement: Supplementary file 1 [file pathogens-14-01076-s001.zip › Supplementary Figure S1.pdf]

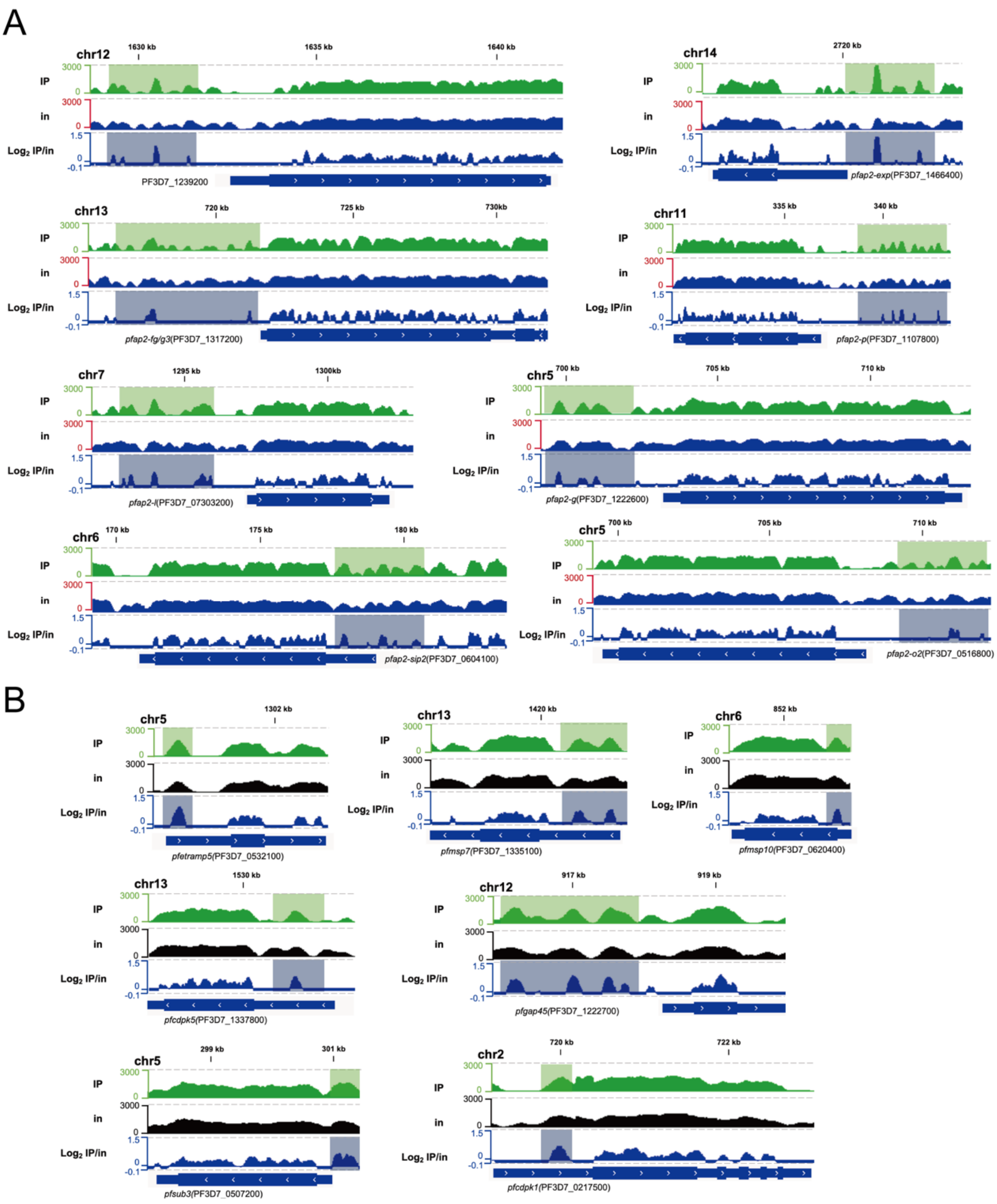

Supplementary Figure S1. PfAP2-06B occupancy at representative apiap2 genes and invasion genes. (A): PfAP2-06B binding *apiap2* genes profiles across the schizont stage[1], including Reads per Kilobase of sequence per Million mapped reads of ChIP, Reads per Kilobase of sequence per Million mapped reads of input, and log2-transformed ChIP/input fold enrichment. (B) PfAP2-06B binding representative invasion genes profiles across the schizont stage[1], including Reads per Kilobase of sequence per Million mapped reads of ChIP, Reads per Kilobase of sequence per Million mapped reads of input, and log2-transformed ChIP/input fold enrichment.

1. Xiaomin S, Xianwen S, Changhong W, Changhong W, Yanting F, Yanting F, Gangqiang G, Gangqiang G, Fei W, Fei W *et al*: **Genome-wide landscape of ApiAP2 transcription**

factors reveals a heterochromatin-associated regulatory network during Plasmodium falciparum blood-stage development. *Nucleic Acids Research* 2022.DOI:10.1093/nar/gkac176
