# Supplementary material for: An ApiAP2 Family Transcriptional Factor PfAP2-06B Regulates Erythrocyte Invasion Indirectly in Plasmodium falciparum
Source: Pathogens. 2025 Oct 22;14(11):1076. doi: 10.3390/pathogens14111076 (PMC12655549; doi:10.3390/pathogens14111076)
Supplement: Supplementary file 1 [file pathogens-14-01076-s001.zip › Supplementary Table S1.pdf]

Table S1 | Primers used for construction and coding sequence

PrimerID	Sequence	Strand
F1	CCTAGTCTAGGGCGCGCCCATATACT<br>TAATATAACCAAAGAAATAACAG	Forward
R1	CCTCGCCCTCATTTCGATAGCTAATTTTTTACTTAAG	Reverse
F2	ATCGAAATGAGGGCGAGGATGACT<br>GGAGAATGTTTAATTTCAG	Forward
R2	GTCAACCGCTGCGGCAGC<br>ATTACTCATAGTTCCTTTTTTTGTTG	Reverse
F3	GCTGCCGCAGCGGTTGACGCAGCA<br>GCAGCTCTCGAG	Forward
R3	TATATTTTATTATTAGCGGCCGCCTATTC<br>CAGTTTCAAAAGTTCGAC	Reverse
F4	GCGGCCGCTAATAATAAAAT	Forward
R4	TTTTACAAAATGCTTAAGTTGCTGGTGCAGTTAATATTG	Reverse
LHR	CATATACTTAATATAACCAAAGAAATAACAGATAATAATAACTTACAT<br>CTTTCAAATAACGAAAAAATAAATAAAAATATTTCATATGAAAATTTA<br>AATATTGATCATACAACAGATGATCTTTTAAAATCATCAGGTAAAGAT<br>ATGAAAGGAGAAACTTTTAATAAACATTATTCAAAAAATTCTTTAAAT<br>AAAAATCTCAAAAAAGAAGTAGATGATGTTAATATGATAAACAAAAT<br>GGAAAAAACAGGATCAACACCTGCAGGAGTATATATGATTAGAATT<br>AATGGTGTTGTACAAGCTTGGAGAGCTGAATGGAGAAGTCCAAGT<br>GGTTGTAAAAGAACAAAAAATTTCGGAATTAATACTTATGGTACTA<br>CCTTAAGTAAAAAATTAGCTATCGAAATGAGgGCgAGgATGACTGG<br>AGAATGTTTAATTTCAGATGATGGAACTGTTTTTGATTATTCAACA<br>AAAAAAGGAACTATGAGTAAT	
2×fkbp-gfp-2×fkbp tag	GCTGCCGCAGCGGTTGACGCAGCAGCAGCTCTCGAGTCAGGATT<br>GAGATCAAGATCTGCTGCTGCTGGTGCTGGTGGTGCTGCTAGAG<br>CTGCTctgcagAGAGGAGTACAAGTTGAAACAATATCACCAGGAGAT<br>GGTCGTACATTTCCAAAAAGAGGTCAAACTTGTGTTGTACATTATAC<br>TGGAATGCTTGAAGATGGAAAGAAATTTGATTCATCTCGTGATAGAA<br>ATAAACCATTTAAATTTATGCTAGGTAAACAAGAAGTAATACGAGGT<br>TGGGAAGAAGGAGTTGCTCAAATGAGTGTAGGTCAAAGAGCAAAAC<br>TTACTATATCTCCAGATTATGCTTATGGTGCAACTGGACATCCAGGTA<br>TAATTCCACCTCATGCAACTCTTGTATTTGATGTGGAGCTTCTAAAAC<br>TAGAAACTAGAGGTGTTCAGGTTGAAACAATTTCACCTGGAGATGGC<br>AGAACCTTTCCTAAAAGAGGACAGACTTGCGTAGTTCATTATACAGGC<br>ATGCTAGAGGATGGTAAGAAATTTGATTCTAGTCGAGATAGAAATAA<br>GCCATTCAAGTTTATGCTAGGTAAACAGGAAGTAATAAGAGGTTGGG<br>AAGAGGGTGTAGCACAGATGTCAGTTGGACAAAGAGCAAAGTTAAC<br>AATATCACCAGATTATGCATACGGTGCAACAGGCCATCCTGGCATCA	

TCCCTCCACATGCAACTTTAGTATTCGACGTTGAATTGTTAAAGTTAG
AGACAacgcgtGCTAGAGGTGCTGCTGCTGGTGCTGGAGGTGCAGGTA
GACGTACGATGAGTAAAGGAGAAGAACTTTTCACTGGAGTTGTCCCA
ATTCTTGTTGAATTAGATGGTGATGTTAATGGGCACAAATTTTCTGTCA
GTGGAGAGGGTGAAGGTGATGCAACATACGGAAAACTTACCCTTAAAT
TTATTTGCACTACTGGAAAACTACCTGTTCCATGGCCAACACTTGTCAC
TACTTTCGCGTATGGTCTTCAATGCTTTGCGAGATACCCAGATCATATG
AAACAGCATGACTTTTTCAAGAGTGCCATGCCCGAAGGTTATGTACAG
GAAAGAACTATATTTTTCAAAGATGACGGGAACTACAAGACACGTGCT
GAAGTCAAGTTTGAAGGTGATACCCTTGTTAATAGAATCGAGTTAAAAG
GTATTGATTTTAAAGAAGATGGAAACATTCTTGGACACAAATTGGAATAC
AACTATAACTCACACAATGTATACATCATGGCAGACAAACAAAAGAATG
GAATCAAAGTTAACTTCAAAATTAGACACAACATTGAAGATGGAAGCGT
TCAACTAGCAGACCATTATCAACAAAATACTCCAATTGGCGATGGCCCT
GTCCTTTTACCAGACAACCATTACCTGTCCACACAATCTGCCCTTTCGA
AAGATCCCAACGAAAAGAGAGACCACATGGTCCTTCTTGAGTTTGTAAC
AGCTGCTGGGATTACACATGGCATGGATGAGCTCTACAAAGTCGACGC
CAGGGGAGCAGCCGCAGGAGCAGGGGGGGCAGGAAGGCGTGGTGTT
CAGGTCGAGACTATTAGCCCTGGAGATGGACGCACGTTTCCTAAGCGT
GGACAGACATGCGTAGTTCACTACACAGGTATGTTGGAGGACGGTAAA
AAGTTCGACAGCTCACGCGACCGCAATAAACCTTTCAAGTTTATGCTTG
GCAAGCAGGAGGTTATTCGTGGATGGGAGGAGGGTGTAGCACAGATG
TCTGTTGGACAGCGTGCTAAGTTGACAATTTCACCTGACTATGCTTATG
GCGCTACGGGCCATCCCGGGATCATTCCGCCACATGCGACTCTGGTA
TTCGACGTTGAATTATTAAAGTTAGAGACAgctagaggggccgctgcaggtgctggtg
gagctggaagaCGTGGAGTACAAGTAGAGACTATCTCTCCAGGTGACGGTC
GCACTTTCCCAAAGCGTGGCCAAACCTGTGTTGTACATTACACTGGTAT
GCTGGAGGATGGGAAAAAGTTCGATTCCAGTCGCGACCGTAACAAACC
GTTCAAATTCATGTTGGGAAAGCAGGAAGTGATCCGCGGGTGGGAGGA
AGGCGTGGCGCAAATGAGCGTCGGTCAGCGGGCTAAATTGACCATTTC
CCCTGACTACGCGTATGGGGCTACTGGGCACCCAGGGATTATTCCGCC
TCACGCTACACTTGTGTTTGATGTCGAACTTTTGAAACTGGAATAG

RHR	GCGGCCGCtaataataaaatatatatacaaaggtgaaattatatcacacttaaatatatatatatatat<br>Atatatatatatatatattaccctctttaaataaaaatttttttaaatattcttttatatatatatatatattatagtgag<br>Ctattatctatatatttaatttaaaaaaaaaaaaaaaaaaaaaaaaatcctgaactgttcaggtaaaatttttt<br>Tagtaatattttacgattttgtctttttttttttttttttttttcttttttttttttttggtaatatatatttaccatataaaataaat<br>Ctttttaaataaattttacattttattttaaaacatctattactattaaaaaataaagaaagaaaaaaaaaaaaa<br>Gtaataaatagataaaaacatttacgcatatatttttccgtttacatatttttttccatttacaatgctggcgttttgaat<br>acaaggatgttatattttttatttttattttgagctgtcattcgtaaatatttattttttacaatattaactgcaccagcaa
sgRNA	GAAATGAGAGCAAGAATGAC
Mutated sgRNA	GAAATGAGgGCgAGgATGAC
