# Supplementary material for: An ApiAP2 Family Transcriptional Factor PfAP2-06B Regulates Erythrocyte Invasion Indirectly in Plasmodium falciparum
Source: Pathogens. 2025 Oct 22;14(11):1076. doi: 10.3390/pathogens14111076 (PMC12655549; doi:10.3390/pathogens14111076)
Supplement: Supplementary file 1 [file pathogens-14-01076-s001.zip › Supplementary Table S2.pdf]

Table S2 | Primers used for verification

PrimerID	Sequence	Strand
P1	GATCCTGTTAAAGTACAGAGAG	Forward
P2	GTGTCTTGTAGTTCCCGT	Reverse
P3	GTGTGATATAATTTCACCTTTGTA	Reverse
