# Supplementary figures and images for: An ApiAP2 Family Transcriptional Factor PfAP2-06B Regulates Erythrocyte Invasion Indirectly in Plasmodium falciparum
Source: Pathogens. 2025 Oct 22;14(11):1076. doi: 10.3390/pathogens14111076 (PMC12655549; doi:10.3390/pathogens14111076)

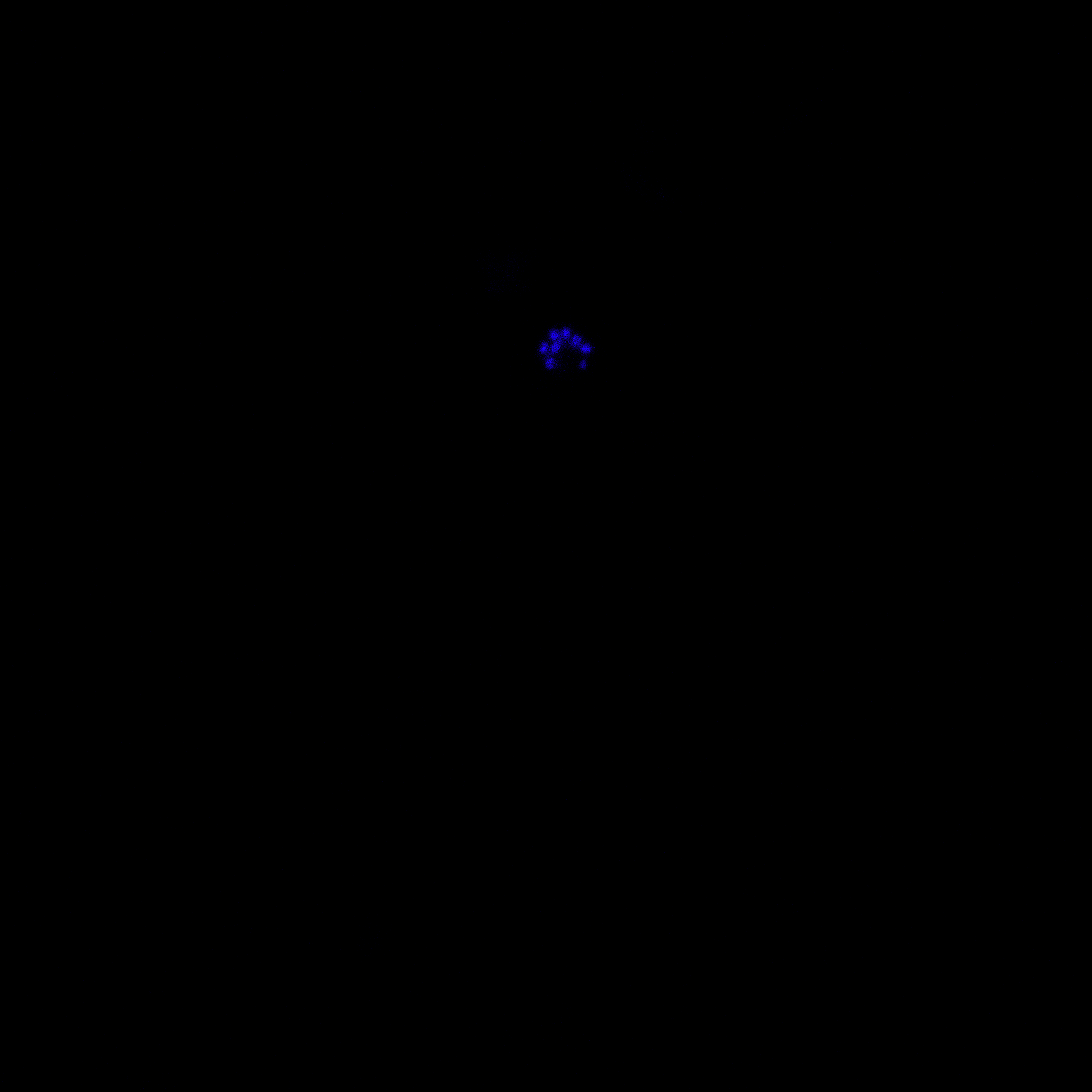

Supplement: Supplementary file 1 [file pathogens-14-01076-s001.zip › Images/EtOH-DAPI.tif]

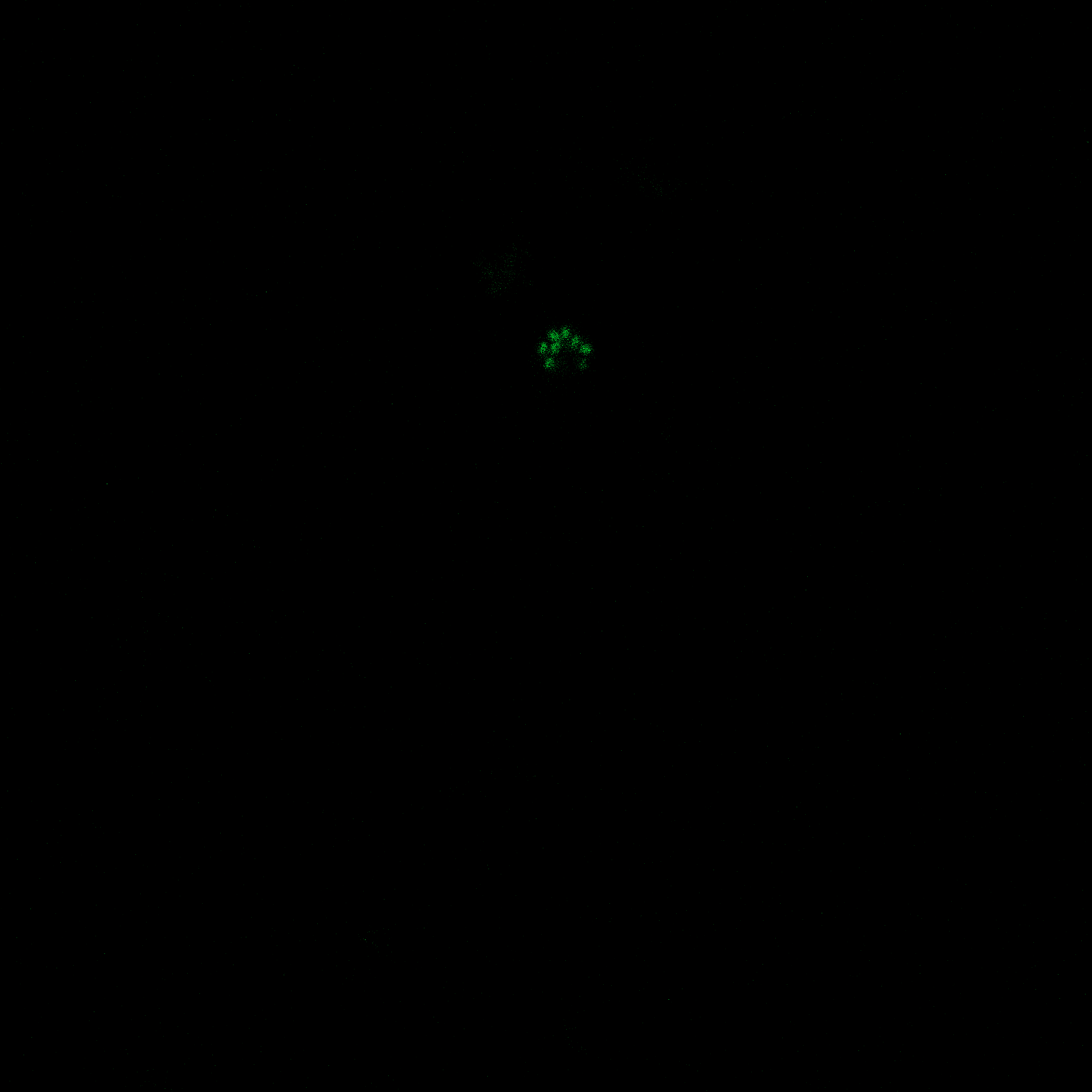

Supplement: Supplementary file 1 [file pathogens-14-01076-s001.zip › Images/EtOH-GFP.tif]

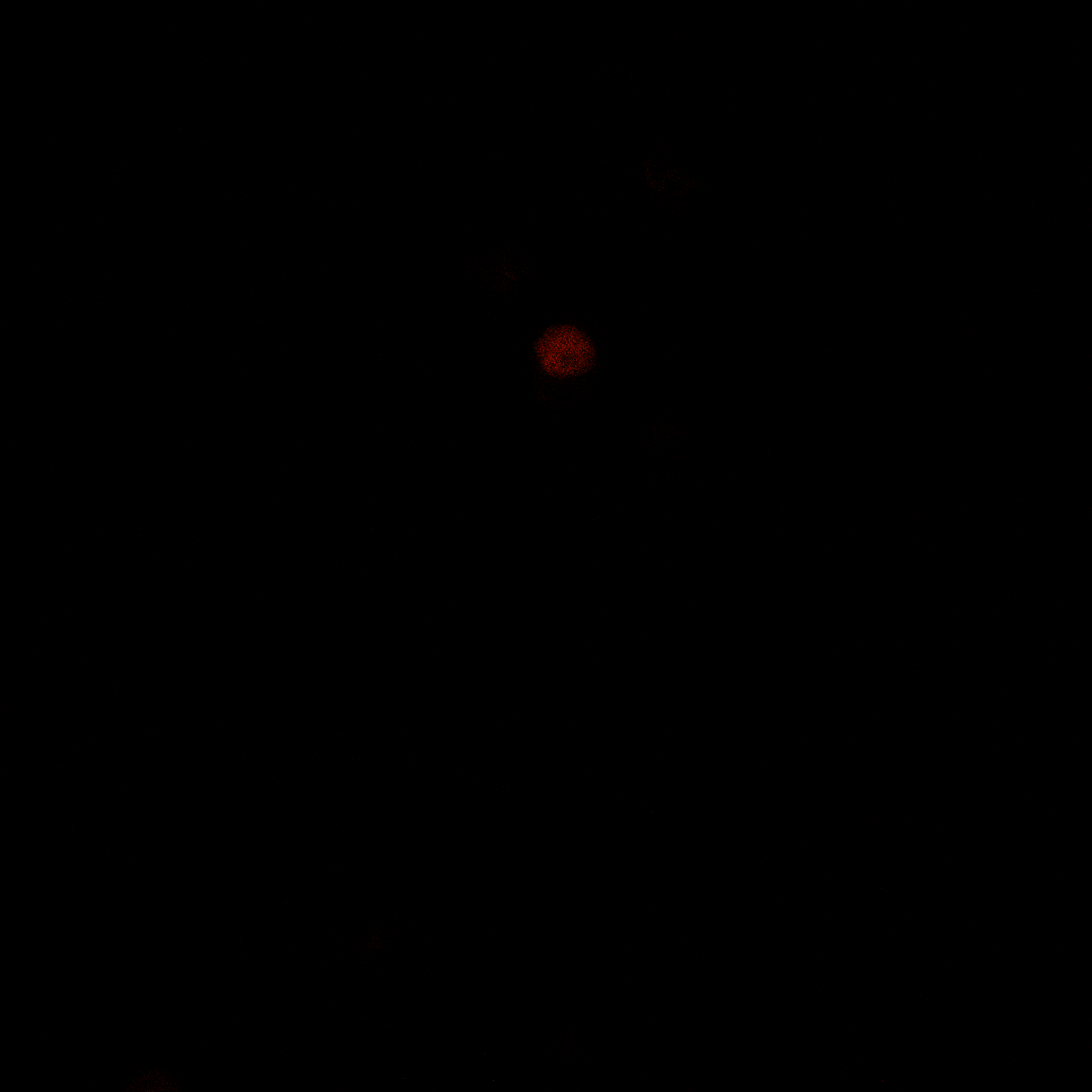

Supplement: Supplementary file 1 [file pathogens-14-01076-s001.zip › Images/EtOH-mCherry.tif]

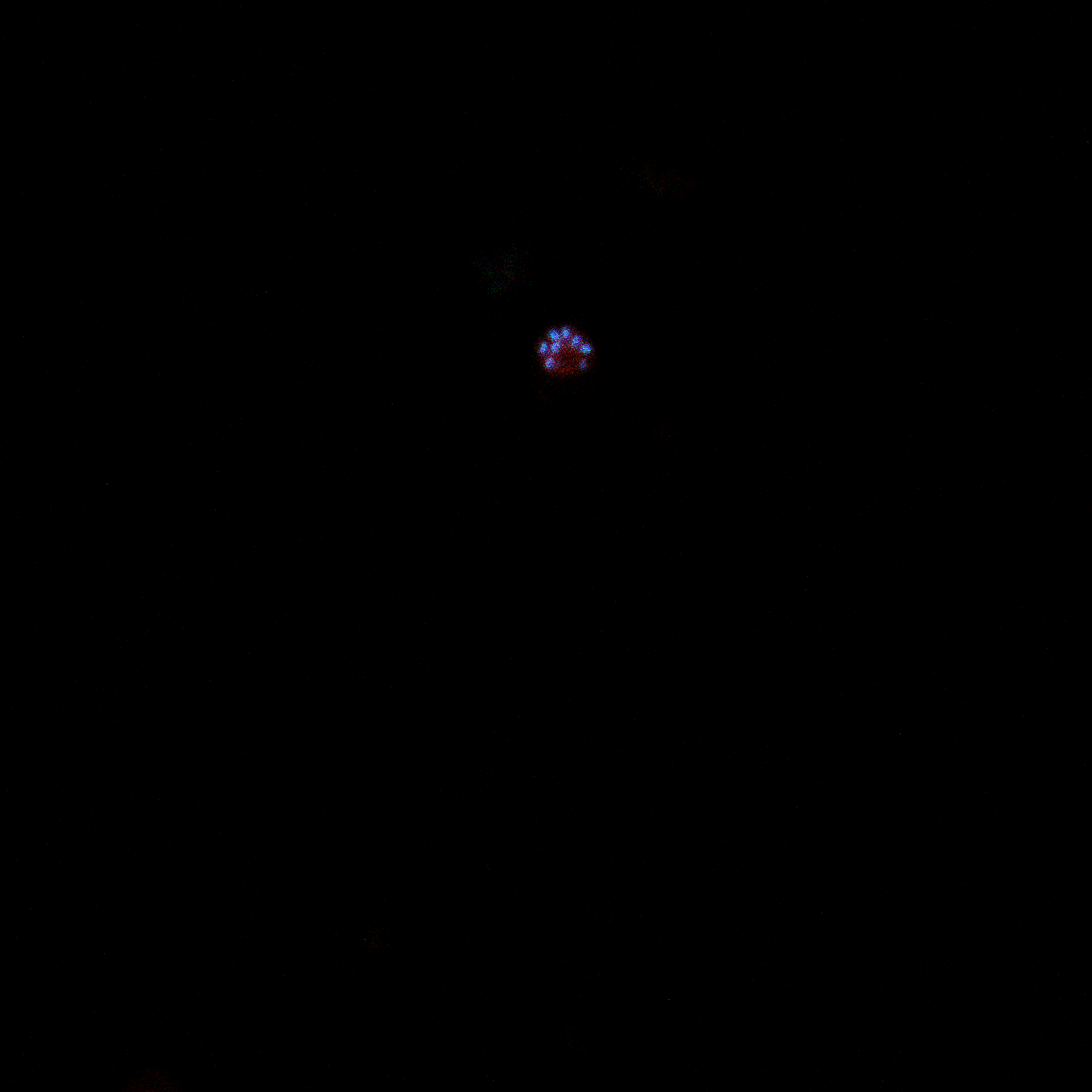

Supplement: Supplementary file 1 [file pathogens-14-01076-s001.zip › Images/EtOH-Merge.tif]

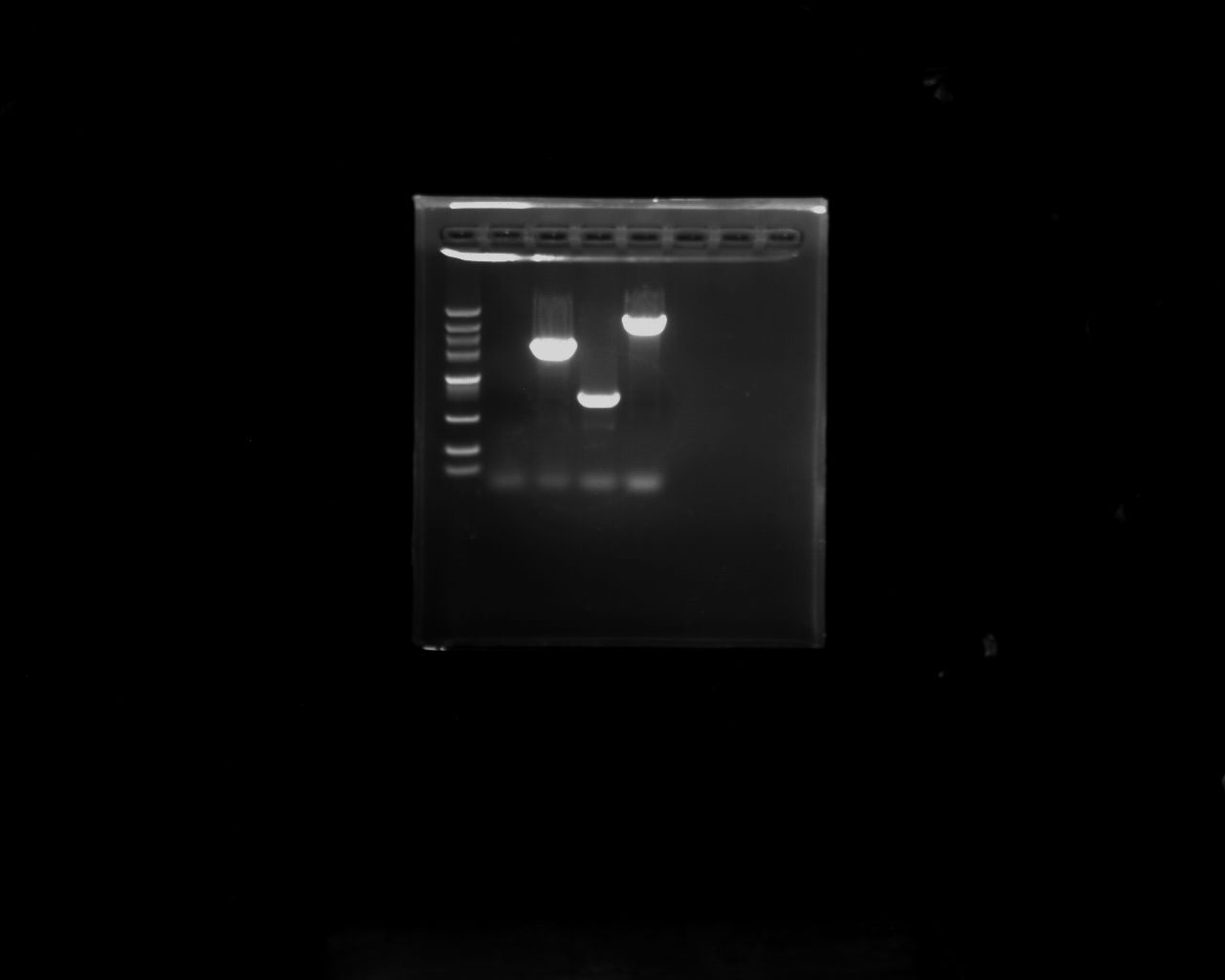

Supplement: Supplementary file 1 [file pathogens-14-01076-s001.zip › Images/Figure 1F.jpg]

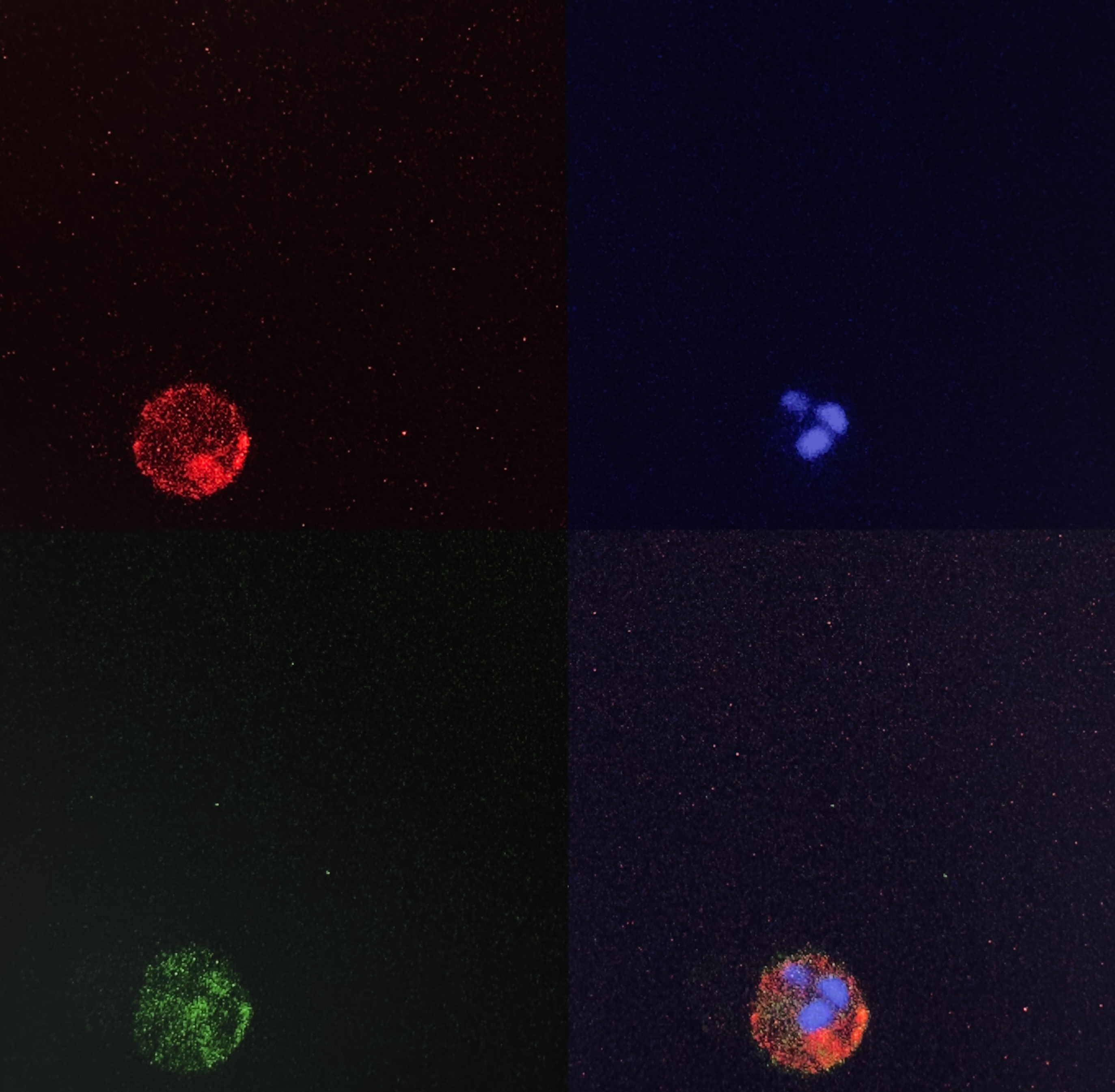

Supplement: Supplementary file 1 [file pathogens-14-01076-s001.zip › Images/Rapa.tif]
